# The COVID-19 pandemic: a threat to antimicrobial resistance containment

**DOI:** 10.2144/fsoa-2021-0012

**Published:** 2021-06-10

**Authors:** Raspail C Founou, Ariel J Blocker, Michel Noubom, Cedrice Tsayem, Siméon P Choukem, Maarten Van Dongen, Luria L Founou

**Affiliations:** ^1^Department of Microbiology, Hematology & Immunology, Faculty of Medicine & Pharmaceutical Sciences, University of Dschang, Dschang, Cameroon; ^2^United COVID-19 TaskForce, University of Dschang, Dschang, Cameroon; ^3^AMR Insights Ambassador Network, Amsterdam, The Netherlands; ^4^Evotec ID Lyon, Severe Bacterial Infections Unit & Bacteriology/Bacteriomics Platform Lyon, France; ^5^District Hospital of Dschang, Dschang, Cameroon; ^6^Department of Clinical Biochemistry, Centre of Expertise & Biological Diagnostic of Cameroon, Yaoundé, Cameroon; ^7^Department of Internal Medicine & Specialties, Faculty of Medicine & Pharmaceutical Sciences, University of Dschang, Dschang, Cameroon; ^8^AMR Insights, Amsterdam, The Netherlands; ^9^Department of Food Safety & Environmental Microbiology, Centre of Expertise & Biological Diagnostic of Cameroon, Yaoundé, Cameroon

**Keywords:** antimicrobial resistance, antimicrobial stewardship, COVID-19 pandemic, COVID-19 vaccines, SARS-CoV-2

## Abstract

As of 23 April 2021, the outbreak of COVID-19 claimed around 150 million confirmed cases with over 3 million deaths worldwide. Yet, an even more serious but silent pandemic, that of antimicrobial resistance (AMR), is likely complicating the outcome of COVID-19 patients. This study discusses the current knowledge on the emergence of the SARS-CoV-2 and highlights the likely contribution of the COVID-19 pandemic on the escalation of AMR. COVID-19 engenders extensive antibiotic overuse and misuse, and will undoubtedly and substantially increase AMR rates worldwide. Amid the expanding COVID-19 pandemic, policymakers should consider the hidden threat of AMR much more, which may well be enhanced through improper use of antibiotics to treat patients with severe COVID-19 infection.

## Lay abstract

Antimicrobial resistance (AMR) is a natural phenomenon that allows microorganisms to resist to the action of antimicrobial medicines that were previously active against them and cured the infection. AMR is caused by the appropriate and inappropriate use of antimicrobial medicines. The occurrence of the COVID-19 pandemic engenders extensive antimicrobial use that is likely to aggravated the AMR pandemic. This paper discusses the current knowledge on the SARS-CoV-2, and underscores the contribution of the COVID-19 pandemic on the escalation of AMR. Beyond the expanding COVID-19 pandemic, the hidden threat of AMR should also be considered by the decision-makers.

An outbreak of COVID-19 caused by SARS-CoV-2 was initially reported in Wuhan, China, in December 2019 [1]. COVID-19 causes principally mild flu-like symptoms such as fever, dry cough and sore throat, with favorable outcomes in the vast majority of cases [2]. Nevertheless, for some 5–10% of the patients, the disease may lead to life-threatening complications such as severe pneumonia, acute respiratory distress syndrome (ARDS), sepsis, septic shock and organ failure [3]. In approximately 50% of cases, such complications lead to death [4]. The multidimensional impacts of COVID-19 prompted the WHO to declare it as a global pandemic on 11 March 2020.

The unprecedented pandemic the world is facing is veiling another even greater and silent pandemic, which is prematurely killing hundreds of thousands of people worldwide and is likely complicating the outcome for COVID-19 patients. This silent pandemic is antimicrobial resistance (AMR), which is further exacerbated by the void in the development of new therapeutic antimicrobial drugs [5,6]. AMR kills, increases the healthcare costs, hampers the control of infectious diseases and has the potential to threaten health security, and weaken trade and economies [7].

People with immune-compromised status together with those already suffering from respiratory tract infections including tuberculosis, influenza, severe acute respiratory syndrome (SARS), and currently COVID-19, are at higher risk of developing secondary infections [8]. In fact, a virus can weaken the immune system and thereby, facilitate secondary difficult-to-treat bacterial or fungal infections that are potentially resistant [9]. The 2009 H1N1 influenza pandemic claimed, for instance, 284,000 deaths worldwide, with over 30% of those being essentially related to a bacterial (co) secondary infection [10]. The spread of SARS-CoV-2 is thus of great concern and could be more severe in low- and middle-income countries (LMICs), where the high burden of infectious diseases coupled with multiple challenges could lead to devastating situations in the already overburdened healthcare systems. The unregulated over-the-counter supplies of antimicrobials in these countries lead to a further increase in AMR. In these resource-constrained countries, the limited availability of diagnostic tools for COVID-19 and weak preparedness to respond to outbreaks of emerging pathogens could further lead to extensive and random antibiotic consumption and even further increase the burden of AMR that is already a major public health threat.

Nevertheless, data on the impact of COVID-19 on AMR are so far limited, a situation that could seriously impede strategies and interventions for AMR containment. Therefore, the purpose of this study was, with what little evidence is presently available, to summarize information regarding the collateral damage of COVID-19 pandemic on AMR containment. By summarizing the available data, our objectives are to: discuss the current knowledge on the emergence of the SARS-CoV-2 and COVID-19, highlight the likely contribution of the COVID-19 pandemic on the further development and spreading of AMR, and delineate prevention and containment strategies for coping with COVID-19 while preserving antimicrobials for future generations.

## Emergence of the SARS-CoV-2 & COVID-19

### SARS-CoV-2 reservoir & transmission pattern

Coronaviruses (CoV) are a group of viruses responsible for up to 30% of flu-like symptoms in humans [11]. The newly emerged SARS-CoV-2 is an enveloped, nonsegmented, single-stranded, positive-sense RNA virus of which the genome has been found to be most closely related with 96.2% identity to a virus detected in bats known as CoV RaTG13, whereas it shares 79.5% identity to SARS-CoV [12]. This suggests that bats are the primary host of the virus, and that the SARS-CoV-2 entered the human population from a spill over event either directly from a bat or through an unknown intermediate host as with the SARS-CoV in 2003 [13].

All human coronaviruses appear to have respiratory transmission, meaning they are pathogens with intrinsically high pandemic potential. The SARS-CoV-2 exhibits a higher degree of transmissibility, and pandemic risk than that of SARS-CoV [14], although the lethality level is lower than that of this virus [12]. Given the pace at which the COVID-19 outbreak has developed, sustained transmission of the SARS-CoV-2 occurs either directly through droplets or person-to-person contact [1], and air [15], or indirectly through fomites [15], and potentially also via the fecal–oral route [16].

Droplet transmission may occur following immediate exposure to respiratory droplets produced during cough or sneezing of a SARS-CoV-2-infected person [14]. Airborne transmission of the SARS-CoV-2 is also plausible [15]. In fact, in analyzing the aerosols (<5 μm) and surface stability of the SARS-CoV-2 and in estimating its decay rates, van Doremalen *et al.* revealed that the SARS-CoV-2 remains viable in aerosols for 1–3 h. Furthermore, the study also shows that the SARS-CoV-2 is stable on plastic, and stainless steel for up to 3 days. The authors thus suggested that aerosols and fomites can be other plausible sources of SARS-CoV-2 transmission [15]. In this instance, a healthy individual might be exposed indirectly via bioaerosols present in the air (i.e., after the infected person has left their proximity), and via contact with contaminated fomites (objects, clothes or surfaces), where the virus could subsequently enter in the body via mucosal surfaces that include mouth, nose or eyes [14]. Although no case of SARS-CoV-2 transmission via the fecal–oral route has yet been intriguingly evidenced, Wu *et al.* demonstrated a 55% prevalence of SARS-CoV-2 RNA in fecal samples of COVID-19-confirmed patients. The authors then surmise that fecal–oral transmission of SARS-CoV-2 should be considered, especially in community settings [16].

### Current status of the COVID-19 pandemic

Since the first reports of cases from China in late 2019, around 150 million confirmed COVID-19 cases and over 3 million deaths were reported worldwide, as of 23 April 2021 [10]. Despite an assortment of systems for pandemic preparedness against emerging and re-emerging pathogens including the WHO International Health Regulations (2005), the high level of COVID-19 confirmed cases so far (as of 23 April 2021) is currently primarily being reported in high-income countries. In the USA, for instance, the world’s most prepared country to deal with a pandemic/epidemic according to the Global Health Security Index [17], around 32 million laboratory confirmed COVID-19 cases were detected as of 23 April 2021, placing the country as most affected globally, ahead of India and Brazil with 16.61 and 14.24 million cases, respectively [10]. The USA further reported the highest number of deaths related to COVID-19 with over 570,000 deaths, ahead of Brazil (386,416 deaths) and India (189,544 deaths) [10]. This suggests that the COVID-19 pandemic threatens all countries regardless of geographic borders and socioeconomic levels.

There is, therefore, an increasing concern about the potential impact of COVID-19 in LMICs, particularly in sub-Saharan African countries, which are still far behind high-resource settings in terms of pandemic preparedness and are thus among the least prepared countries with significant gaps to address [10]. LMICs have so far reported relatively lower confirmed COVID-19 cases and deaths compared with high-income countries [10]. While it is unclear whether the low reports of COVID-19 cases and deaths in these countries are due to a lack of testing, related to an earlier stage of the epidemic or due to other factors, cases are however escalating in several LMICs and emerging economies [10].

Of serious concern, the WHO warned that more cases of COVID-19 are likely to be reported in resource-constrained countries, and that widespread transmission of SARS-CoV-2 will happen in the upcoming months, including extensive community spread [17]. SARS-CoV-2 should spread significantly in LMICs where 69% of the global population aged of ≥60 live, the impact of COVID-19 could be devastating given the already low-resourced health systems and other challenges such as cramped living conditions, poor access to clean drinking water, suboptimal hygiene and sanitation, malnutrition, current pandemic (including tuberculosis and HIV/AIDS), endemic (malaria) and epidemic infectious diseases (cholera, measles and Ebola, etc.) [18].

A widespread outbreak of SARS-CoV-2 in LMICs will not only impact their health systems and populations, but would likely have significant societal and economic impacts worldwide. The COVID-19 pandemic has led to large numbers of people in need of medical care at the same time, resulting to overloaded public and private healthcare settings and overwhelmed healthcare workforce facing elevated rates of hospitalizations and deaths [19]. Additionally, the world economic uncertainty engendered by the COVID-19 pandemic, will cost up to $2 trillion in 2020 alone globally [19]. Global strategies and interventions to monitor the SARS-CoV-2 in order to inspect and lessen its public health and socioeconomic impacts are thus urgently needed as the COVID-19 pandemic will considerably affect the world, especially LMICs [18].

## Emergence of SARS-CoV-2 variants & COVID-19 vaccines

### SARS-CoV-2 variants

As with other viruses, SARS-CoV-2 naturally undergoes mutations during their evolution. Several variants of SARS-CoV-2 have been detected across the world. These have been classified as variants of interest (VOI), variants of concern (VOC) and variants of high consequence (VOHC) by the CDC (Table 1) [20]. Notwithstanding, three new variants including B.1.1.7 (also known as VOC-202012/01), 501Y.V2 (B.1.351) and P.1 (B.1.1.28.1) have rapidly spread to become predominant within their countries and across the world [21].

**Table 1.** List of COVID-19 vaccines currently approved for emergency use in at least one country.

N	Primary developers	Origin	Vaccine name	Vaccine type	Phase	Status
1	Pfizer, BioNTech, Fosun Pharma	Multinational	Comirnaty (BNT162b2)	mRNA-based vaccine	II, III	Approved in several countries and by the WHO. Emergency use in the USA, EU, other countries
2	Moderna, BARDA, NIAID	USA	Moderna COVID-19 Vaccine (mRNA-1273)	mRNA-based vaccine	III	Approved in Switzerland. Emergency use in the USA, EU, other countries
3	BARDA, OWS	UK	COVID-19 vaccine AstraZeneca (AZD1222) also known as Vaxzevria and Covishield	Adenovirus vaccine	II, III	Approved in Brazil. Stopped use in Denmark. Emergency use in UK, EU, other countries
4	Gamaleya Research Institute, Acellena Contract Drug Research and Development	Russia	Sputnik V	Recombinant adenovirus vaccine (rAd26 and rAd25)	III	Early use in Russia. Emergency use in other countries
5	Janssen Vaccines (Johnson & Johnson)	The Netherlands, USA	COVID-19 vaccine Janssen (JNJ-78436735; Ad26.COV2.S)	Nonreplicating viral vector	III	Emergency use in the USA, EU, other countries. Paused in some states and countries
6	Beijing Institute of Biological Products; China National Pharmaceutical Group (Sinopharm)	China	BBIBP-CorV	Inactivated vaccine	III	Approved in China, UAE, Bahrain. Emergency use in other countries
7	Sinovac Biotech Co., Ltd.	China	CoronaVac	Inactivated vaccine (formalin with alum adjuvant)	III	Approved in China. Emergency use in other countries
8	Federal Budgetary Research Institution State Research Center of Virology and Biotechnology	Russia	EpiVacCorona	Peptide vaccine	III	Approved in Russia. Emergency use in other countries
9	CanSino Biologics	China	Convidicea (Ad5-nCoV)	Recombinant vaccine (adenovirus type 5 vector)	III	Approved in China. Emergency use in other countries
10	Bharat Biotech, ICMR	India	Covaxin (BBV152)	Inactivated vaccine	III	Emergency use in India, other countries
11	Wuhan institute of Biological Products, China National Pharmaceutical Group (Sinopharm)	China	WIBP-CorV	Inactivated vaccine	III	Approved in China. Limited use in UAE
12	Novavax	USA	NVX-CoV2373	Protein	III	Approved in several countries and by the WHO

The B.1.1.7 variant with its 23 mutations leading to 17 amino acid changes was first described in the UK on 14 December 2020 [22]. Similarly, the 501Y.V2 (B.1.351) variant displaying 23 mutations with 17 amino acid changes was initially detected in South Africa on 18 December 2020 [23], while the P.1 variant exhibiting around 35 mutations with 17 amino acid changes was reported in Brazil on 12 January 2021 [24]. These three variants share the N501Y mutation while two additional receptor-binding-domain mutations, K417N/T and E484K were detected in the 501Y.V2 and P.1 variants. These mutations increase the binding affinity of the ACE2 receptor [21]. As such, the 501Y.V2 variant has been showed to be 50% more transmissible than previous variants in South Africa [25], while B.1.1.7 transmissibility is estimated to range from 43 to 82% compared with that of variants preexisting in the UK [26]. Two other SARS-CoV-2 variants, B.1.427 and B.1.429, recently detected in California and classified as VOC, have been reported to be 20% more transmissible than preexisting variants [21]. Hence, four important concerns arising from the emerging SARS-CoV-2 variants are their effects on: viral transmissibility, reinfection rates (i.e., escape from natural immunity), disease severity and vaccine effectiveness (i.e., escape from vaccine-induced immunity). These concerns lead to three main questions: how will existing COVID-19 vaccines and vaccine candidates cope with emerging SARS-CoV-2 variants? Will vaccination modification be needed to respond to new variants? How well will the vaccines perform against COVID-19 infections including mild and moderate cases?

### COVID-19 vaccines

The pace in the development of a vaccine against the COVID-19 has been so fast that within less than 12 months after the beginning of the COVID-19 pandemic, several research teams rose to the challenge and developed vaccines that effectively protect against SARS-CoV-2. Two vaccines namely BNT162b2 from PfiZer-BioNTech and Moderna COVID-19 Vaccine (mRNA-1273) from Moderna, both using mRNA-based vaccine platform achieved regulatory authorization for emergency use from the US FDA in December 2020 [27,28].

As of today, up to 12 COVID-19 vaccines (Table 1) received emergency regulatory authorization from the FDA or approval from at least one country with seven being actively rolled out throughout the world as part of the COVAX initiative that aims to provide global equitable access to COVID-19 vaccines at low cost [29]. Most of these COVID-19 vaccines appear to be effective and safe albeit more research is needed to investigate not only the long-term efficacy and safety of these vaccines but more so, the influence on the containment of AMR. For instance, the BNT162b2 vaccine displays a 95% of vaccine efficacy [30]. A recent study with real-world data from Israel further confirmed that this vaccine is highly effective in preventing COVID-19 [31]. However, there is currently a lack of data on how well this vaccine and other will work at preventing COVID-19 with SARS-CoV-2 variants, especially the B.1.1.7 SARS-CoV-2. While it is suggested that the majority of COVID-19 vaccines might contend reasonably well against B.1.1.7, the B.1.351 variant is a cause of considerable concern for which the vaccine efficacy seems to be lower.

All these vaccine strategies are directed toward the viral spike protein, but mutation occurring in the *S* gene of SARS-CoV-2 variants threatens their continued efficacy. The term vaccine resistance has been used by some scientist to describe the reduced efficacy of COVID-19 vaccines against some variants. A recent study from Hacisuleyman *et al.* reported two cases of breakthrough infection in a cohort of 417 persons having received their second dose of mRNA-1273 (Moderna, MA, USA) or BNT162b2 (Pfizer–BioNTech) vaccine at least 2 weeks earlier [32]. Notwithstanding proof of vaccine efficacy, both patients developed symptoms of COVID-19 and were tested positive for SARS-CoV-2 by PCR. Genome sequencing revealed mutations of potential clinical importance including E484K in one and three mutations (T95I, del142–144 and D614G) in both patients [32]. These observations reveal a likely risk of symptomatic COVID-19 upon successful vaccination and ensuing infection with SARS-CoV-2 variants. They further provide evidences for continuous efforts to prevent and diagnose infection as well as to characterize SARS-CoV-2 variants in vaccinated persons [32].

## The contribution of COVID-19 to the development & escalation of AMR

### Evidence of antibiotic misuse & overuse in the current COVID-19 pandemic

Antibiotic resistance, as part of the broader AMR, is the ability of bacteria to grow in the presence of antibiotics that are normally active against them. Antibiotic use is the key factor in the development of antibiotic resistance, with community and hospital settings being the equal ecological niches of its emergence in human health [5,6]. In fact, the selective pressure exerted whenever an antibiotic is used, either rationally or irrationally, contributes to the selection of resistant bacteria. Upon their selection, resistant bacteria can survive in an organism for at least a year [33] and can spread directly from person-to-person or indirectly via the food chain and the environment [5,6].

The COVID-19 pandemic severely challenges all aspects of healthcare including both the diagnosis and treatment of bacterial infections and effective delivery of antibiotics. Given that patients with viral pneumonia such as COVID-19 are typically at increased risk of developing severe secondary bacterial infections, it is indubitable that, especially in the often-hectic conditions, antibiotics are being empirically prescribed in many COVID-19 patients. This assertion has been confirmed in a handful of published reported, although mainly from Chinese hospitals, which revealed that antibiotics are being extensively administered in COVID-19 patients. In fact, a recent study revealed that antivirals and antibiotics were used in 93 and 100% of ICU and non-ICU patients, respectively [34]. The study further showed that only four (10%) out of 41 patients had a secondary infection, suggesting thereby a 90% rate of antibiotic misuse. Similarly, in a retrospective cohort study, Zhou *et al.* showed that 95, 98 and 93% of all patients, nonsurvivors and survivors, respectively, received antibiotics. In staggering contrast, antiviral treatment was given only to 21% of patients, with 22 and 21% being given to nonsurvivors and survivors, respectively [4]. The authors further revealed that 50% of nonsurvivor patients experienced secondary (bacterial) infections [4]. Similar findings were evidenced by Chen *et al.*, who showed that 71% of patients received an antibiotic treatment, with 45% of these being treated with a combination therapy as a prevention against common pathogens. Bacteria were laboratory confirmed in merely 1% of patients with *Klebsiella pneumoniae, Acinetobacter baumannii* and *Aspergillus flavus* being isolated in one patient [35]. The study further showed that this *A. baumannii* displayed high resistance to antibiotics [35]. The antibiotic inefficacy may thus be related to resistant bacteria although it is plausible that initiation and duration of antibiotic treatment in each patient has significantly affected the outcomes, as would have their co-morbidities. Likewise, in a meta-analysis, Clancy *et al.* revealed that bacterial lung superinfections were the cause of deaths in 32% of COVID-19-positive cases globally [36]. The authors further showed that 79% of patients were treated with antibacterial agents and the leading etiologic agents were *A. baumannii*, *P. aeruginosa*, *K. pneumoniae*, *E. coli* and *S. aureus* [36].

These studies reveal at sufficiency the highly undesirable apparent inappropriate antibiotic treatment in patients with COVID-19. Considering that COVID-19 is caused by a virus that leads to a self-limiting infection in the majority of cases, antibiotic treatment implemented in COVID-19 patients, especially when secondary bacterial infections are not laboratory confirmed, are indicative of a well-recognized global pattern of antibiotic misuse that has contributed to AMR emergence and spread [6]. Although it is currently uncertain whether COVID-19 or the ICU treatments it requires contribute to secondary bacterial infections, it is urgent that secondary infections such as bacterial pneumoniae in particular, be carefully ascertained before antibiotic administration. This is particularly important to reduce the misuse and overuse of antibiotics, and the subsequent emergence and escalation of AMR, across countries or regions while taking care of COVID-19 patients. This calls for a much wider use of bacterial identification and antibiotic susceptibility testing prior to antibiotic prescription.

Promising therapeutic medications are increasingly being approved for large-scale experimental studies. Consequently, several pharmaceutical products have been suggested as potential investigational therapies. Results generated through several preliminary *in vitro* and *in vivo* clinical studies revealed, for instance, that chloroquine, an old and well-described antimalarial drug, and especially hydroxychloroquine, one of its derived forms, could be effective in the control of SARS-CoV-2 as monotherapy or in combination with azithromycin [37,38]. However, the studies of Gautret *et al.* in particular, where, for instance, patient numbers were initially very small and an untreated study arm is still blatantly missing are currently highly controversial. Yet given the current absence of any COVID-19-specific therapeutics, the combination of hydroxychloroquine and azithromycin has since been extensively used compassionately in Italy, for instance, albeit without any firm evidence of clinical efficacy.

This bears the risk that azithromycin, a broad-spectrum macrolide antibiotic, which is primarily used in the treatment of respiratory, enteric and sexually transmissible infections caused by an assortment of extra- and intracellular pathogens such as *Streptococcus pneumoniae*, *Haemophilus influenzae*, *Salmonella spp.*, *Neisseria gonorrheae*, among others [39] will be massively administered for the COVID-19 management. The overuse of azithromycin, first-line antibiotic in the management of pneumonia, in the treatment of the COVID-19 patients will likely result in sustained selection of resistant bacteria coupled with increased resistance not only to this antibiotic but also the macrolide family of drugs more broadly and, through coselection due to the fact that resistance genes are often carried together on multidrug resistance plasmids, to other antibiotic families within the patients’ gut and respiratory microbiomes, contributing thereby to exponential emergence and spread of AMR [40].

Furthermore, the chloroquine family of drugs is also used to treat other diseases such as lupus and the present increased worldwide demand for these drugs is causing shortages for these chronically ill patients currently [41]. Overdosing is also causing any increase in prevalence of the known most dangerous side effects of these drugs, such as cardiac arrhythmia [42]. Finally, and perhaps most worrisomely, chloroquine family of drugs is still widely used worldwide to treat malaria [43]. This means that if the combination hydroxychloroquine and azithromycin is used to treat COVID-19 patients in tropical areas of the planet where *Plasmodium* species are also still prevalent, in particular without prior testing for coinfection, there is a serious risk of increasing also the already widespread resistance of these deadly parasites to this family of drugs and related antimalarial drugs.

The concern of AMR will further be exacerbated in LMICs where over-the-counter supply of and counterfeit antibiotics prevail, and AMR prevention and containment measures are limited or nonexistent [44]. It is thus hypothesized that antibiotics will not only be extensively used in hospitals but also in community settings in these resource-constrained countries. Consequently, further studies investigating the risk of AMR in relation to the COVID-19 pandemic are urgently needed in order to strengthen knowledge about the multidimensional impacts of this pandemic and improve patients’ outcomes while preserving the efficacy of antibiotics and achieving sustainable development.

## Collateral damage of COVID-19 on AMR emergence & spread

Human coronavirus including the SARS-CoV-2 is one of the main pathogens involved in respiratory tract infections. There are significant similarities between the COVID-19 and AMR pandemics. Both are responsible for high infection-related morbidity and mortality worldwide. The COVID-19 shares similar clinical and radiological features to that of a bacterial respiratory tract infection, and diagnosis and treatment of people with secondary, possibly resistant, bacterial infections and/or COVID-19 are likely to be compromised as all attention currently focuses on the treatment of the COVID-19 [9,45]. We postulate that increased pressure exerted on health systems by the COVID-19 pandemic, will impede people with resistant infections from access to rapid and accurate diagnostic and treatment, resulting in adverse outcomes. Vulnerable populations including elderly, people with long-term use of immunosuppressive agents, pregnant women and those with co-morbidities are at higher risk of developing negative outcomes in both diseases. Adequate investigation of secondary bacterial infections in these patients is thus imperative to improve their outcome because if resistant pathogens cause a secondary infection in COVID-19 patients, then the management of both infections becomes harder [45].

**Figure 1.**
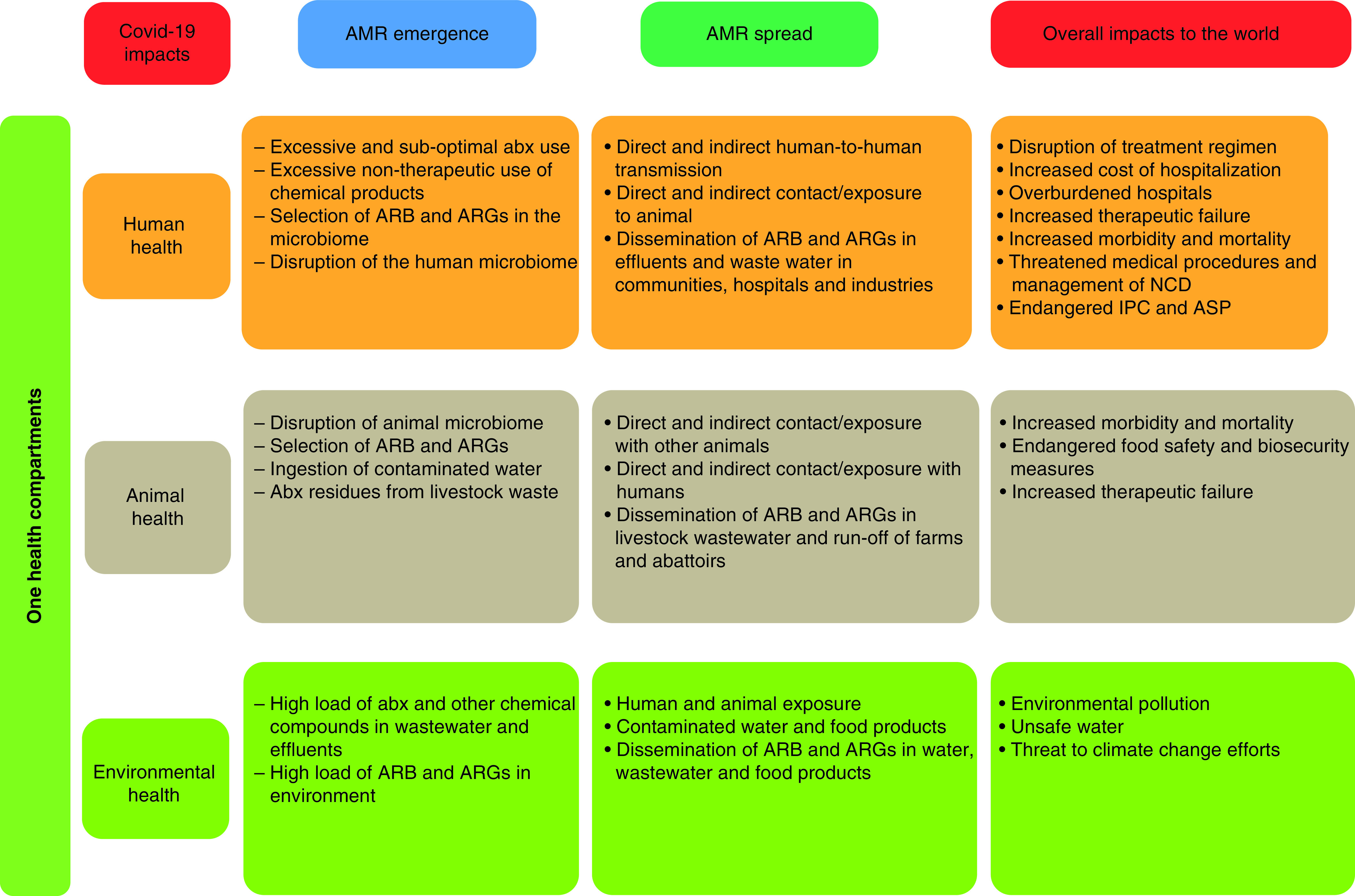
Summary of COVID-19 impacts on antimicrobial resistance emergence and spread. abx: antibiotics; AMR: Antimicrobial resistance; ARB: Antibiotic-resistant bacteria; ARG: Antibiotic resistance gene; ASP: Antimicrobial stewardship program; IPC: Infection, prevention and control; NCD: Noncommunicable disease.

We hypothesize that AMR is therefore a greater threat hidden behind the COVID-19 pandemic that may not only further complicate outcomes of many hospitalized COVID-19 patients but also has consequences that are likely to be more severe and far-reaching than that of the COVID-19 pandemic (Figure 1). In fact, AMR is projected to become the leading cause of mortality worldwide with 10 million deaths yearly and cost up to US$ 100 trillion of the global economy loss if considerable efforts are not sustainably implemented by 2050 [5,46]. The World Bank further confirmed these estimates, and showed that AMR will lead to a global increase in healthcare costs ranging from $300 billion to more than $1 trillion by 2030 with LMICs being the most affected both in terms of mortality rates and economic shortfalls and around 24 million of people forced into extreme poverty [47]. However, all these speculations did not consider nor envision the unforeseen emergence of a virus that will cause a pandemic with severe multidimensional implications.

It has also been predicted that AMR outcomes could be prevented with a moderate investment of $42 billion [5,46]. Specifically, it was estimated that US$16 billion were needed to revamp the antimicrobials research and development pipeline using new market incentives. This cost was modeled on achieving at least four breakthrough products targeting bacterial species of greatest concern within a decade [45]. Moreover, US$2 billion over 5 years are needed to set up an AMR Global Innovation Fund, while US$3–4 billion a year are needed to take global action such as improved water and sanitation as well as good general disease surveillance [45].

Given that the COVID-19 pandemic has the potential to engender extensive antibiotic overuse and misuse, it will undoubtedly and substantially increase AMR rates worldwide. Furthermore, this increases risks being especially rapid and deleterious in areas, often in LMICs such as India, South Africa but also in Italy and Spain, for instance, where prevalence of multidrug resistance plasmids is already high [48,49]. Thereby, the COVID-19 pandemic may allow AMR to reach future estimates earlier than that was predicted.

## Challenges of COVID-19 pandemic in the containment of AMR

COVID-19 disrupts effectiveness of well-established antimicrobial stewardship (AMS) programs as well as AMR surveillance research that have been deprioritized, delayed or halted with alteration of health system resources as part of the pandemic response [50]. In fact, resources, personnel and attention were redirected from AMR containment strategies to COVID-19 response including diagnosis, contact tracking and tracing [50] despite the fact that AMR surveillance data are essential to help researchers and physicians to better monitor evolution of resistance patterns and impact of antibiotic use linked to COVID-19 and secondary infections and coinfections. This emphasizes failures in AMR containment and mitigation strategies that necessitate urgent attention from scientific and clinical communities. These include assessment of the global incidence of secondary infections and coinfections in COVID-19 patients to identify patients most in need of antibiotic treatment as well as when the treatment can be safely discontinued, de-escalated or withheld [50].

COVID-19 has further evidenced how vulnerable the world and especially the healthcare systems are. This is even more visible and severe in LMICs that usually lack infrastructures and qualified personnel and are also not well prepared to deal with pandemic situations [50]. Sustainable global surveillance of societal and clinical antibiotic consumption and resistance trends is essential to anticipate subsequent changes in AMR epidemiology while preventing antimicrobial shortages and ensuring continuous supply. These strategies make a case for implementation research on AMR and represent implementation challenges in LMICs.

## Conclusion

The COVID-19 pandemic is highly interrelated, multisectoral and there are no geographic or income boundaries to contain it. Amid the expanding COVID-19 pandemic, governments and policymakers do have to consider the hidden threat of AMR, which, at present, is jeopardizing health, societal and economic advancements globally, in their endeavor to manage the current COVID-19 crisis. The targeted, rational treatment of secondary bacterial infections should be an integral part of pandemic planning. Mobilization of substantial funding for research, and better monitored antibiotic stewardship programs as well as limiting the utterly over-the-counter supply of antibiotics are urgently needed to ensure containment of the COVID-19 and AMR.

## Future perspective

**Figure 2.**
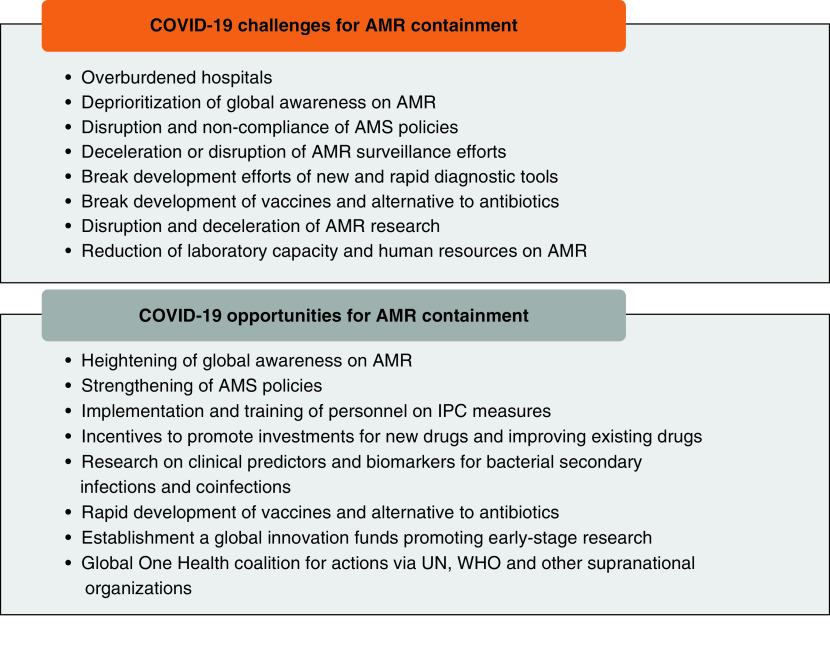
Summary of COVID-19 challenges and opportunities for antimicrobial resistance containment. AMR: Antimicrobial resistance; AMS: Antimicrobial surveillance; IPC: Infection, prevention and control; UN: United Nations.

Although existing weaknesses in the containment of AMR have been magnified by the COVID-19 pandemic, there are several opportunities that can be seized from the current situation and could affect the effective containment of AMR. This section briefly describes key recommendations and opportunities for a strong research agenda and sustainable containment of AMR during and beyond this pandemic (Figure 2).

First, prospective studies must be standardized in term of data being collected, population investigated and microbiological sampling and analyses. Research on clinical predictors and biomarkers for bacterial secondary infections and coinfections should be envisioned at hospital admission. Global surveillance of resistance must be strengthened in both COVID-19 and non-COVID-19 patients with foremost bacteria, antibiotic resistance genes and mobile genetic elements being recorded along with adequate indicators on a regular basis [50]. Epidemiological data originating from patients colonized or infected with high-risk bacteria should also be collected. High-risk patients and wards (ICUs, hematological wards) must be actively monitored and continuously screened for colonization of high-risk bacteria.

Second, assessing the correlation between changes in antibiotic consumption during the pandemic and escalation of AMR must be implemented along with analysis of global AMR surveillance data, especially from LMICs, this in order to understand likely emerging resistance patterns and the driver forces behind them.

Third, a multidisciplinary approach in line with the One Health, must be implemented in hospitals and communities as it is the case in the management of COVID-19 pandemic. AMS programs should not only remain active but more so be reinforced with activities also targeting noninfectious disease physicians.

Finally, as key component in the COVID-19 pandemic response, global awareness and political will on AMR should also be heightened for an effective and sustainable containment of this silent threat. In fact, communication between governments, the media, researchers, healthcare professionals and the public has been a key component in the management of COVID-19 [51]. The AMR scientific community thus plays a key role and is at the heart of this strategy favoring collaboration with policymakers and the media to raise AMR awareness to the general population and build on community awareness and engagement such as the observed social distancing, handwashing, disinfections or wearing of mask measures [52]. The COVID-19 pandemic has evidenced the multidimensional repercussions of an uncontrolled infectious disease, a situation that is comparable to what has already been predicted for AMR. Assessing the effects of antibiotic use, disruption of AMS, while strengthening infection, prevention and control measures, and global awareness and political will on AMR is critical for an effective AMR containment in the future and over a longer timescale. Multisectoral, coordinated and targeted research on AMR and in line with the One Health approach is required for containment of the challenges arising from AMR in the context of COVID-19 pandemic or upcoming threats.

**Executive summary**

- Since the first reports of cases from China in late 2019, around 150 million confirmed COVID-19 cases and over 3 million deaths were reported worldwide as of 23 April 2021.
- The unprecedented pandemic the world is facing is veiling another even greater and silent pandemic that is already prematurely killing hundreds of thousands of people worldwide and is likely complicating the outcome for COVID-19 patients, that of antimicrobial resistance (AMR).
- The COVID-19 pandemic is highly interrelated, multisectoral and there are no geographic or income boundaries to contain it.
- This pandemic has evidenced the multidimensional repercussions of an uncontrolled infectious disease, a situation that is comparable to what has already been predicted for AMR.
- The consequences of the AMR pandemic are likely to be even more severe, long-term and far-reaching than that of the COVID-19 pandemic.
- Despite the gaps evidenced, data on the impact of COVID-19 on AMR are far limited, a situation that could seriously impede strategies and interventions for AMR containment.
- Assessing the effects of antibiotic use, disruption of AMS, while strengthening infection, prevention and control measures, and global awareness and political will on AMR is critical for an effective AMR containment in the future and over a longer timescale.
- The current uptake of routine vaccination against SARS-CoV-2 although reducing the impact of this pandemic is endangered with emerging variants.
- Although existing weaknesses in the containment of AMR have been magnified by the COVID-19 pandemic, there are several opportunities that can be seized from the current situation and could affect the effective containment of AMR.

## References

[1] World Health Organization (WHO). Report of the WHO-China Joint Mission on Coronavirus Disease 2019 (COVID-19). WHO, Geneva, Switzerland (2020).

[2] Gralinski LE, Menachery VD. Return of the coronavirus: 2019-nCoV. Viruses 12(2), 135 (2020). 10.3390/v12020135 PMC7077245 31991541

[3] Wang J, Du G. COVID-19 may transmit through aerosol. Irish J. Med. Sci. 189(4), 1143–1144 (2020). 32212099 10.1007/s11845-020-02218-2 PMC7094991

[4] Zhou F, Yu T, Du R Clinical course and risk factors for mortality of adult inpatients with COVID-19 in Wuhan, China: a retrospective cohort study. Lancet 395(10229), 1054–1062 (2020). 32171076 10.1016/S0140-6736(20)30566-3 PMC7270627

[5] O’Neill J. Antimicrobial resistance: tackling a global health crisis: initial steps. Rev. Antimicrob. Resist. (2015).

[6] Founou LL, Founou RC, Essack SY. Antibiotic resistance in the food chain: a developing country-perspective. Front. Microbiol. 7(1881), (2016). 10.3389/fmicb.2016.01881 PMC5120092 27933044

[7] Lai C-C, Shih T-P, Ko W-C, Tang H-J, Hsueh P-R. Severe acute respiratory syndrome coronavirus 2 (SARS-CoV-2) and coronavirus disease-2019 (COVID-19): the epidemic and the challenges. Int. J. Antimicrob. Agents 55(3), 105924 (2020). 32081636 10.1016/j.ijantimicag.2020.105924 PMC7127800

[8] Gerberding JL. Antibiotic resistance: the hidden threat lurking behind Covid-19. : STAT. Globe Media, MA, USA (2020). https://www.statnews.com/2020/03/23/antibiotic-resistance-hidden-threat-lurking-behind-covid-19/

[9] Morris DE, Cleary DW, Clarke SC. Secondary bacterial infections associated with influenza pandemics. Front. Microbiol. 8, 1041–1041 (2017). 28690590 10.3389/fmicb.2017.01041 PMC5481322

[10] Roser M, Ritchie H, Ortiz-Ospina E. Coronavirus pandemic (COVID-19). https://ourworldindata.org/coronavirus

[11] Weston S, Frieman MB. COVID-19: knowns, unknowns, and questions. mSphere 5(2), e00203–00220 (2020). 32188753 10.1128/mSphere.00203-20 PMC7082143

[12] Guo Y-R, Cao Q-D, Hong Z-S The origin, transmission and clinical therapies on coronavirus disease 2019 (COVID-19) outbreak – an update on the status. Military Med. Res. 7(1), 11 (2020). 10.1186/s40779-020-00240-0 PMC7068984 32169119

[13] Adhikari SP, Meng S, Wu Y-J Epidemiology, causes, clinical manifestation and diagnosis, prevention and control of coronavirus disease (COVID-19) during the early outbreak period: a scoping review. Infect. Dis. Poverty 9(1), 29 (2020). 32183901 10.1186/s40249-020-00646-x PMC7079521

[14] van Doremalen N, Bushmaker T, Morris DH Aerosol and surface stability of SARS-CoV-2 as compared with SARS-CoV-1. New Engl. J. Med. 382, 1564–1567 (2020). 32182409 10.1056/NEJMc2004973 PMC7121658

[15] Wu Y, Guo C, Tang L Prolonged presence of SARS-CoV-2 viral RNA in faecal samples. Lancet Gastroenterol. Hepatol. 5(5), 434–435 (2020). 32199469 10.1016/S2468-1253(20)30083-2 PMC7158584

[16] Nuclear Threat Initiative, The Johns Hopkins Center for Health Security, The Economist Intelligence Unit. Global Health Security Index: Building Collective Action and Accountability. (2019). https://www.ghsindex.org/

[17] Lloyd-Sherlock P, Ebrahim S, Geffen L, McKee M. Bearing the brunt of COVID-19: older people in low and middle income countries. BMJ 368, m1052 (2020). 32169830 10.1136/bmj.m1052

[18] Essack SY, Bell J, Burgoyne DS, Duerden M, Ther D, Shephard A. Topical (local) antibiotics for respiratory infections with sore throat: an antibiotic stewardship perspective. J. Clin. Pharm. Ther. 44(6), 829–837 (2019). 31407824 10.1111/jcpt.13012 PMC6899613

[19] United Nations Conference on Trade and Development (UNCTAD). The Coronavirus Shock: A Story of Another Global Crisis Foretold and What Policymakers Should be Doing About It. UNCTAD, NY, USA (2020).

[20] Center for Disease Control and Prevention (CDC). SARS-CoV-2 variant classifications and definitions. (2021). https://www.cdc.gov/

[21] Abdool Karim SS, de Oliveira T. New SARS-CoV-2 variants – clinical, public health, and vaccine implications. New Engl. J. Med. 384,1866–1868 (2021). 33761203 10.1056/NEJMc2100362 PMC8008749

[22] Public Health England. Investigation of novel SARS-COV-2 variant: variant of concern 202012/01. (2020). https://www.gov.uk/government/organisations/public-health-england

[23] Tegally H, Wilkinson E, Giovanetti M Detection of a SARS-CoV-2 variant of concern in South Africa. Nature 592(7854), 438–443 (2021). 33690265 10.1038/s41586-021-03402-9

[24] Voloch CM, da Silva Francisco R Jr, de Almeida LGP Genomic characterization of a novel SARS-CoV-2 lineage from Rio de Janeiro, Brazil. J. Virol. 1,JVI.00119–21 (2021). 10.1128/JVI.00119-21 PMC8139668 33649194

[25] Pearson CAB, Russell TW, Davies N Estimates of severity and transmissibility of novel SARS-CoV-2 variant 501Y.V2 in South Africa. Centre for the Mathematical Modelling of Infectious Diseases (CMMID) Repository (2021).

[26] Davies NG, Abbott S, Barnard RC Estimated transmissibility and impact of SARS-CoV-2 lineage B.1.1.7 in England. Science 372(6538), eabg3055 (2021). 33658326 10.1126/science.abg3055 PMC8128288

[27] Moderna I. U.S. FDA Authorizes mRNA Vaccine Against COVID-19 for Emergency Use. Moderna, Cambridge, UK (2020).

[28] Pfizer-BioNTech. Vaccines and Related Biological Products Advisory Committee meeting December 10, 2020. FDA Briefing Document Pfizer-BioNTech COVID-19 Vaccine. (2020). https://www.fda.gov

[29] World Health Organization (WHO). What is the Access to COVID-19 Tools (ACT) accelerator, how is it structured and how does it work? WHO, Geneva, Switzerland (2021).

[30] Polack FP, Thomas SJ, Kitchin N Safety and efficacy of the BNT162b2 mRNA Covid-19 vaccine. New Engl. J. Med. 383(27), 2603–2615 (2020). 33301246 10.1056/NEJMoa2034577 PMC7745181

[31] Dagan N, Barda N, Kepten E BNT162b2 mRNA Covid-19 vaccine in a nationwide mass vaccination setting. New Engl. J. Med. 384(15), 1412–1423 (2021). 33626250 10.1056/NEJMoa2101765 PMC7944975

[32] Hacisuleyman E, Hale C, Saito Y Vaccine breakthrough infections with SARS-CoV-2 variants. New Engl. J. Med. (2021). 10.1056/NEJMoa2105000 PMC8117968 33882219

[33] Huang C, Wang Y, Li X Clinical features of patients infected with 2019 novel coronavirus in Wuhan, China. Lancet 395(10223), 497–506 (2020). 31986264 10.1016/S0140-6736(20)30183-5 PMC7159299

[34] Chen N, Zhou M, Dong X Epidemiological and clinical characteristics of 99 cases of 2019 novel coronavirus pneumonia in Wuhan, China: a descriptive study. Lancet 395(10223), 507–513 (2020). 32007143 10.1016/S0140-6736(20)30211-7 PMC7135076

[35] Gautret P, Lagier J-C, Parola P Hydroxychloroquine and azithromycin as a treatment of COVID-19: results of an open-label non-randomized clinical trial. Int. J. Antimicrob. Agents 56(1), 105949 (2020). 32205204 10.1016/j.ijantimicag.2020.105949 PMC7102549

[36] Clancy CJ, Schwartz IS, Kula B, Nguyen MH. Bacterial superinfections among persons with coronavirus disease 2019: a comprehensive review of data from postmortem studies. Open Forum Infect. Dis. 8(3), (2021). 10.1093/ofid/ofab065 PMC7928570 33732753

[37] Yao X, Ye F, Zhang M, Cui C, Huang B, Niu P. *In vitro* antiviral activity and projection of optimized dosing design of hydroxychloroquine for the treatment of severe acute respiratory syndrome coronavirus 2 (SARS-CoV-2). Clin. Infect. Dis. 71(15), 732–739 (2020). 32150618 10.1093/cid/ciaa237 PMC7108130

[38] McMullan BJ, Mostaghim M. Prescribing azithromycin. Aust. Prescr. 38(3), 87–89 (2015). 26648627 10.18773/austprescr.2015.030 PMC4653965

[39] Mack I, Sharland M, Berkley JA, Klein N, Malhotra-Kumar S, Bielicki J. Antimicrobial resistance following azithromycin mass drug administration: potential surveillance strategies to assess public health impact. Clin. Infect. Dis. 70(7), 1501–1508 (2020). 31633161 10.1093/cid/ciz893 PMC7670997

[40] Rainsford KD, Parke AL, Clifford-Rashotte M, Kean WF. Therapy and pharmacological properties of hydroxychloroquine and chloroquine in treatment of systemic lupus erythematosus, rheumatoid arthritis and related diseases. Inflammopharmacology 23(5), 231–269 (2015). 26246395 10.1007/s10787-015-0239-y

[41] Saussine A, Loriot MA, Picard C Chloroquine cardiotoxicity in long-term lupus therapy in two patients. Ann. Dermatol. Venereol. 136(6–7), 530–535 (2009). 19560616 10.1016/j.annder.2009.01.016

[42] Mwanza S, Joshi S, Nambozi M The return of chloroquine-susceptible Plasmodium falciparum malaria in Zambia. Malar. J. 15(1), 584 (2016). 27919256 10.1186/s12936-016-1637-3 PMC5139104

[43] Ndihokubwayo JB, Yahaya AA, Desta AT Antimicrobial resistance in the African region: issues, challenges and actions proposed. Afr. Health Monitor. (16), (2013).

[44] Seaton RA. Antibiotic prescribing in the context of COVID-19 pandemic. British Society for Antimicrobial Chemotherapy, Birmingham, UK (2020).

[45] O’Neill J. Tackling drug-resistant infections globally: final report and recommendations. Rev. Antimicrob. Resist. (2016).

[46] World Bank. Drug-resistant infections: a threat to our economic future (discussion draft). World Bank, Washington, DC, USA (2016).

[47] Mbelle NM, Feldman C, Sekyere JO, Maningi NE, Modipane L, Essack SY. Pathogenomics and evolutionary epidemiology of multi-drug resistant clinical *Klebsiella Pneumoniae* isolated from Pretoria, South Africa. Sci. Rep. 10(1), 1232 (2020). 31988374 10.1038/s41598-020-58012-8 PMC6985128

[48] Nithya N, Remitha R, Jayasree PR, Faisal M, Manish Kumar PR. Analysis of beta-lactamases, bla(NDM-1) phylogeny & plasmid replicons in multidrug-resistant* Klebsiella* spp. from a tertiary care centre in south India. Indian J. Med. Res. 146(Suppl.), S38–S45 (2017). 10.4103/ijmr.IJMR_31_16 PMC5735569 29205194

[49] Oliva M, Monno R, D’Addabbo P A novel group of IncQ1 plasmids conferring multidrug resistance. Plasmid 89, 22–26 (2017). 27916622 10.1016/j.plasmid.2016.11.005

[50] Rodríguez-Baño J, Rossolini GM, Schultsz C Key considerations on the potential impacts of the COVID-19 pandemic on antimicrobial resistance research and surveillance. Trans. R. Soc. Trop. Med. Hyg. (2021). 10.1093/trstmh/trab048 PMC8083707 33772597

[51] Wang H, Cleary PD, Little J, Auffray C. Communicating in a public health crisis. Lancet Digit. Health 2(10), e503–e503 (2020). 32838253 10.1016/S2589-7500(20)30197-7 PMC7417177

[52] Collignon P, Beggs JJ, Walsh TR, Gandra S, Laxminarayan R. Anthropological and socioeconomic factors contributing to global antimicrobial resistance: a univariate and multivariable analysis. Lancet Planet Health 2(9), e398–e405 (2018). 30177008 10.1016/S2542-5196(18)30186-4

